# Serum Metabolomics Uncovers the Mechanisms of Inulin in Preventing Non-Alcoholic Fatty Liver Disease

**DOI:** 10.3390/ph17070895

**Published:** 2024-07-05

**Authors:** Yunhong Sun, Wenjun Zhou, Mingzhe Zhu

**Affiliations:** 1 School of Public Health, Shanghai University of Traditional Chinese Medicine, Shanghai 201203, China; syh_010122@163.com; 2 Institute of Digestive Diseases, Shanghai University of Traditional Chinese Medicine, Shanghai 200032, China

**Keywords:** non-alcoholic fatty liver disease, inulin, metabolomics, metabolites

## Abstract

Inulin may be a promising therapeutic molecule for treating non-alcoholic fatty liver disease (NAFLD). However, the underlying mechanisms of its therapeutic activity remain unclear. To address this issue, a high-fat-diet-induced NAFLD mouse model was developed and treated with inulin. The NAFLD phenotype was evaluated via histopathological analysis and biochemical parameters, including serum levels of alanine aminotransferase, aspartate aminotransferase, liver triglycerides, etc. A serum metabolomics study was conducted using ultra-performance liquid chromatography coupled with tandem mass spectrometry. The results revealed that inulin mitigated NAFLD symptoms such as histopathological changes and liver cholesterol levels. Through the serum metabolomics study, 347 differential metabolites were identified between the model and control groups, and 139 differential metabolites were identified between the inulin and model groups. Additionally, 48 differential metabolites (such as phosphatidylserine, dihomo-γ-linolenic acid, L-carnitine, and 13-HODE) were identified as candidate targets of inulin and subjected to pathway enrichment analysis. The results revealed that these 48 differential metabolites were enriched in several metabolic pathways such as fatty acid biosynthesis and cardiolipin biosynthesis. Taken together, our results suggest that inulin might attenuate NAFLD partially by modulating 48 differential metabolites and their correlated metabolic pathways, constituting information that might help us find novel therapies for NAFLD.

## 1. Introduction

Non-alcoholic fatty liver disease (NAFLD) is the most common chronic liver disease, with an estimated global prevalence of 25% [1]. The spectrum of NAFLD ranges from the benign form of non-alcoholic fatty liver disease to the severe form of non-alcoholic steatohepatitis (NASH), which may progress to fibrosis, cirrhosis, and even liver cancer [2]. The estimated annual medical costs directly attributed to NAFLD treatment exceed USD 100 billion in the United States; moreover, the prevalence of NAFLD has been increasing in children and young adolescents [3]. Because of its widespread prevalence and increased economic burden, NAFLD has become a critical public health issue globally; therefore, it is crucial to investigate and develop efficient therapeutic strategies for NAFLD.

However, the pathogenesis of NAFLD is very complex. NAFLD treatment mainly includes lifestyle intervention, antioxidant therapy, and lipid metabolism regulation, and currently, there are no efficient drugs approved for clinical use for treating NAFLD [4]. Hence, it is important to explore complementary and alternative methods to prevent NAFLD. Inulin is a natural, plant-derived storage polysaccharide with a wide variety of food and pharmaceutical applications [5]. Inulin can promote the proliferation of probiotics, inhibit the growth of intestinal spoilage bacteria, improve the intestinal microenvironment, regulate blood glucose levels, reduce body weight, lower blood lipid levels, and inhibit the expression of inflammatory factors [6]. Inulin supplementation has been shown to accelerate body weight loss in obese mice by increasing the levels of the gut commensal bacterium *Alistipes* and indole-3-acrylic acid [7]. To date, several studies have also reported the efficacy of inulin in treating NAFLD. A randomized, double-blind, placebo-controlled trial showed that a brief treatment with metronidazole followed by inulin supplementation could reduce alanine aminotransferase (ALT) levels in patients with NAFLD [8]. Another study reported that the synbiotic combination of probiotics and inulin could reduce NAFLD severity by modulating the gut microbiota [9]. Yang et al. found that inulin intake alleviated hepatic steatosis, likely by regulating the gut microbiota composition and function and restoring intestinal barrier integrity [10]. A previous study also showed that inulin treatment effectively prevented liver steatosis in a fat-enriched diet-induced NAFLD mouse model, probably by remodeling the intestinal microbiota composition [11]. A literature search revealed that most studies on the treatment of NAFLD using inulin focused on changes in the gut microbiota. However, the mechanisms through which inulin could prevent NAFLD may be very complex and require further elucidation.

Metabolomics is a rapidly evolving omics technology developed after the introduction of genomics, transcriptomics, and proteomics; it involves the high-throughput detection of all small-molecule metabolites in biological fluids, cells, and tissues, and it has been widely used for investigating physiological and pathophysiological processes [12]. Untargeted metabolomics performed using liquid chromatography–mass spectrometry (LC-MS) and gas chromatography–mass spectrometry (GC-MS) is currently the most commonly used metabolomics research tool, and it has been used to screen quantitative details of known and unknown metabolites to reveal key metabolites and their associations with diseases [13,14]. For example, Wen et al. used ultra-performance liquid chromatography coupled with quadrupole/time-of-flight mass spectrometry (UPLC-QTOF/MS) untargeted metabolomics analysis to detect intestinal metabolites in children with Henoch–Schönlein Purpura (HSP). The authors found that the biosynthesis and metabolism of unsaturated fatty acids, particularly arachidonic acid and its metabolites, might be involved in the occurrence and development of HSP [15]. Jia et al. used untargeted metabolomics to reveal the molecular mechanisms of COVID-19 and identified metabolic reprogramming of glucose metabolism and the urea cycle as potential pathological mechanisms of COVID-19 [16].

In the present study, we developed a high-fat-diet-induced NAFLD mouse model and conducted LC-MS untargeted serum metabolomics to determine the underlying mechanisms of the prevention of NAFLD by inulin. The findings may help to identify novel approaches for treating NAFLD.

## 2. Results

### 2.1. Inulin Augmented Biochemical Indices in NAFLD

**Figure 1:**
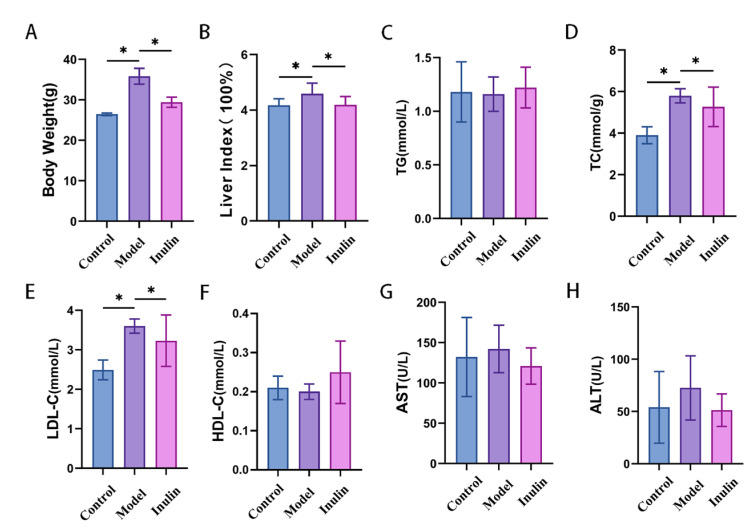
Biochemical indices of mice in each group. (**A**) Body weight, (**B**) liver index, (**C**) serum TG, (**D**) serum TC, (**E**) serum LDL-C, (**F**) serum HDL-C, (**G**) serum AST, and (**H**) serum ALT. Data are expressed as means ± SD (n = 9; * *p* < 0.05).

As shown in Figure 1, compared to the control group, the model group showed a significant increase in body weight, the ratio of liver weight to body weight, and serum levels of total cholesterol (TC) and low-density lipoprotein cholesterol (LDL-C) (*p* < 0.05). Moreover, although the serum levels of aspartate aminotransferase (AST) and ALT were elevated in the model group, the difference was not significant when compared with those of the control group (*p* > 0.05). Notably, inulin partially restored changes in body weight, the ratio of liver weight to body weight, and serum levels of TC, LDL-C, AST, and ALT. However, the serum triglyceride (TG) and high-density lipoprotein cholesterol (HDL-C) levels were not significantly changed among the control, model, and inulin groups (*p* > 0.05).

### 2.2. Inulin Restored Histopathological Changes Caused by NAFLD

**Figure 2:**
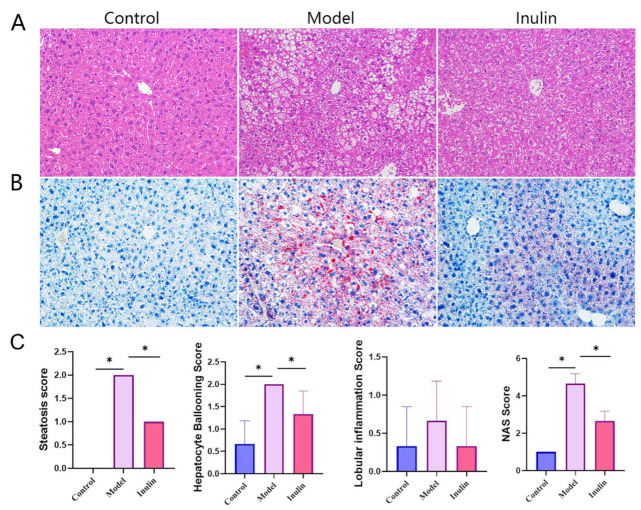
Histopathological changes in liver tissues of mice in each group. (**A**) H&E staining of liver tissues (magnification, ×200). (**B**) ORO staining of liver tissues (magnification, ×200). (**C**) NAFLD activity score (NAS Score), including steatosis, inflammation, and ballooning scores (* *p* < 0.05).

To observe the histopathological changes caused by NAFLD in the liver, liver tissues were stained with hematoxylin and eosin (H&E) and Oil Red O (ORO). H&E staining (Figure 2A) and the NAS score (Figure 2C) revealed that steatosis and hepatocyte ballooning were significantly higher in the model group than in the control group, and these processes were partially restored by inulin (*p* < 0.05). The levels of inflammation among the control, model, and inulin groups exhibited no significant differences (*p* > 0.05). ORO staining (Figure 2B) showed no apparent lipid droplets in the control group, a large number of red lipid droplets in the model group, and a large reduction in the area of red lipid droplets in the inulin group.

### 2.3. Multivariate Statistical Analysis of Serum Metabolites

**Figure 3:**
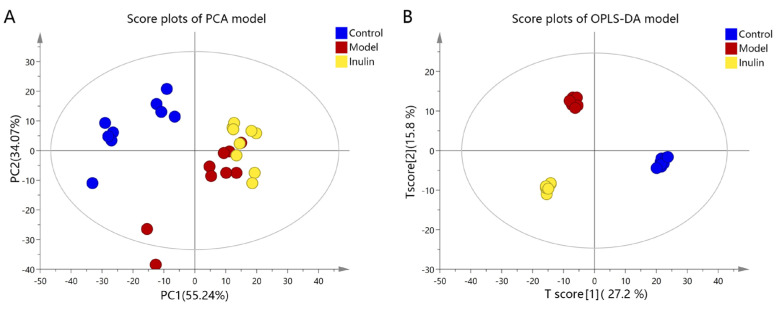
PCA and OPLS-DA plots among the three groups. (**A**) PCA score plots of the control, model, and inulin groups. Blue, red, and yellow dots represent the control, model, and inulin groups, respectively. (**B**) OPLS-DA score plots of the control, model, and inulin groups. Blue, red, and yellow dots represent the control, model, and inulin groups, respectively.

**Figure 4:**
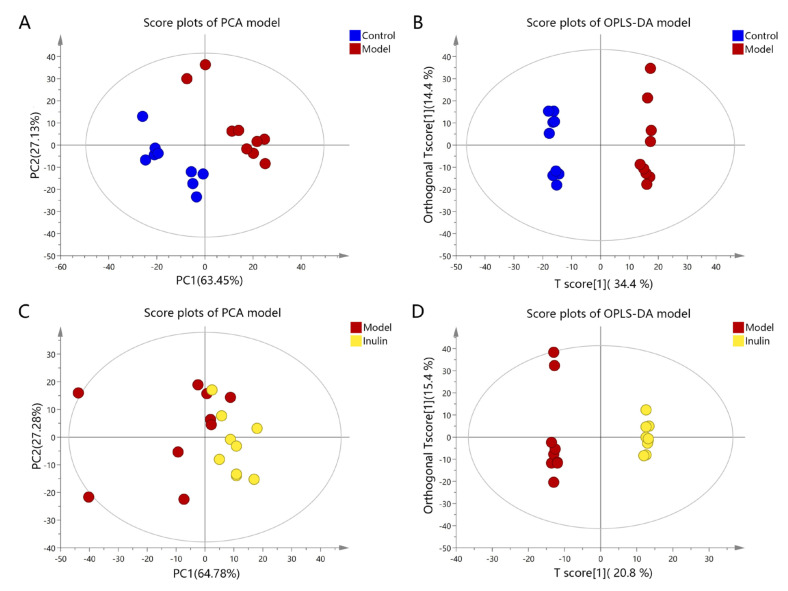
PCA and OPLS-DA plots between pairwise groups. (**A**) PCA score plots of the control and model groups. Blue and red dots represent the control and model groups, respectively. (**B**) OPLS-DA score plots of the control and model groups. Blue and red dots represent the control and model groups, respectively. (**C**) PCA score plots of the inulin and model groups. Red and yellow dots represent the model and inulin groups, respectively. (**D**) OPLS-DA score plots of the inulin and model groups. Red and yellow dots represent the model and inulin groups, respectively.

**Figure 5:**
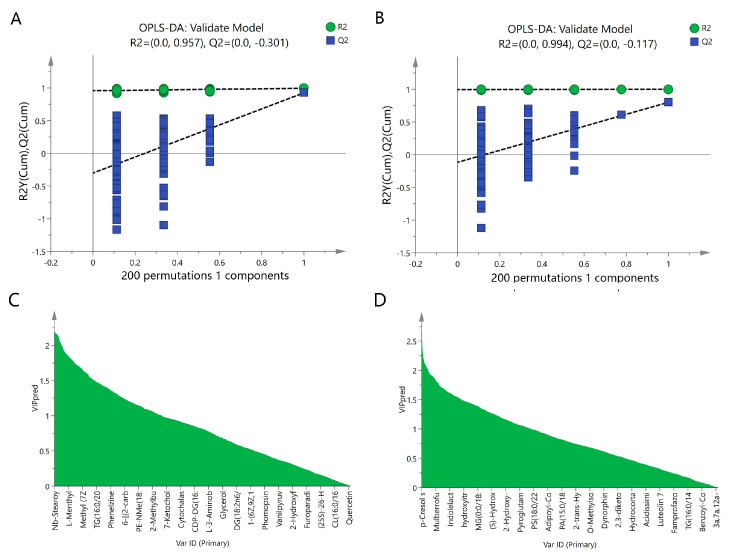
Validation models of OPLS-DA and VIP values. (**A**) The validation model of OPLS-DA for the model and control groups. Green dots and blue squares represent R^2^ and Q^2^, respectively. (**B**) The validation model of OPLS-DA for the inulin and model groups. Green dots and blue squares represent R^2^ and Q^2^, respectively. (**C**) VIP values of OPLS-DA for the model and control groups. X-axis represents metabolite names, and Y-axis represents VIP values. (**D**) VIP values of OPLS-DA for the inulin and model groups. X-axis represents metabolite names, and Y-axis represents VIP values.

To reveal the mechanisms through which inulin prevents NAFLD, a metabolomics study was conducted to obtain metabolite profiles and identify differential metabolites in liver tissue samples. First, a multivariate statistical analysis was performed using SIMCA 14.1 software. As shown in Figure 3A, principal component analysis (PCA) revealed that the metabolites among the control, model, and inulin groups exhibited a clustering trend with R^2^X(cum) = 0.541 and Q^2^ (cum) = 0.159. Orthogonal partial least squares discriminant analysis (OPLS-DA) revealed that the metabolites among three groups exhibited significant separation, with R^2^X(cum) = 0.534, Q^2^(cum) = 0.836, and CV-ANOVA *p* < 0.05 (Figure 3B). PCA and OPLS-DA were also performed between pairwise groups (the control group vs. the model group and the inulin group vs. the model group). PCA analysis revealed there was clustering trend of metabolites between the control and model groups, with R^2^X(cum) = 0.576 and Q^2^ (cum) = 0.185 (Figure 4A), and OPLS-DA analysis revealed a significant separation between the control and model groups, with R^2^X(cum) = 0.459, Q^2^(cum) = 0.926, and CV-ANOVA *p* < 0.05 (Figure 4B). Furthermore, PCA analysis also revealed there was clustering trend between the inulin and model groups, with R^2^X(cum) = 0.539 and Q^2^(cum) = 0.069 (Figure 4C), and OPLS-DA analysis also revealed a significant separation between the inulin and model groups, with R^2^X(cum) =0.478, Q^2^ (cum) = 0.801, and CV-ANOVA *p* < 0.05 (Figure 4D). To validate the OPLS-DA model against overfitting, permutation tests were performed 200 times. As shown in Figure 5A, the OPLS-DA model for the control and model groups exhibited good reliability, with R^2^ = (0.0, 0.957) and Q^2^ = (0.0, −0.301). Figure 5B also shows that the OPLS-DA model for the inulin and model groups had good reliability, with R^2^ = (0.0, 0.994) and Q^2^ = (0.0, −0.117). The variable importance projection (VIP) values of metabolites between the control and model groups and between the model and inulin groups are shown in Figure 5C and 5D, respectively.

### 2.4. Inulin Restored Changes in Metabolites in NAFLD

**Figure 6:**
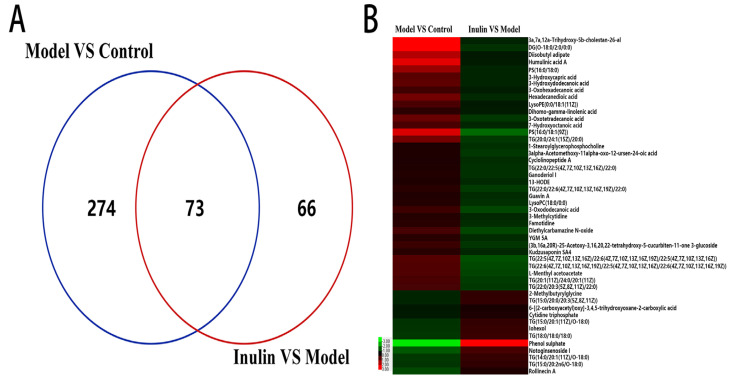
Venn diagram and hierarchical cluster heatmap. (**A**) Venn diagram comparing the two pairwise groups (model vs. control and inulin vs. model) revealed 73 overlapped metabolites. (**B**) A hierarchical cluster heatmap of 48 metabolites whose changes were reversed following inulin treatment. Red and green indicate an increase and decrease in the levels of metabolites.

To identify the candidate metabolites of inulin that played a role in preventing NAFLD, differential metabolites were obtained between pairwise groups, with threshold values of VIP > 1 and *p* < 0.05. A total of 347 differential metabolites were noted between the model and control groups, and there were 139 differential metabolites between the inulin and model groups. Supplementary Tables S1 and S2 present detailed information on these differential metabolites, respectively. The Venn diagram reveals 73 overlapped differential metabolites (Figure 6A and Supplementary Table S3) between the pairwise groups (model vs. control and inulin vs. model). Among these 73 differential metabolites, changes in 48 differential metabolites (Figure 6B and Table 1) were reversed by inulin treatment; these metabolites were identified as candidate metabolites of inulin in preventing NAFLD. For instance, the levels of phosphatidylserine (PS), dihomo-γ-linolenic acid (DGLA), and 13-HODE (13-hydroxyloctadecadienoic acid) were increased in the model group compared to those in the control group and reduced in the inulin group compared to those in the model group. The L-carnitine levels were lower in the model group than in the control group and increased following inulin treatment.

### 2.5. Inulin Altered Metabolic Pathways Affected by NAFLD

**Figure 7:**
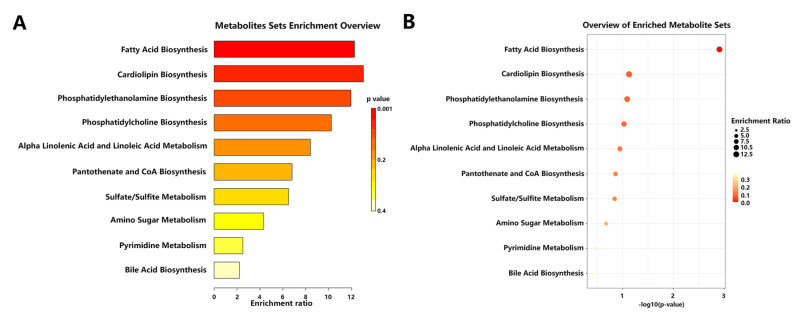
Overview of metabolic pathway enrichment analysis. (**A**) Overview of top 25 enriched pathways. The X-axis represents the enrichment ratio, and the Y-axis represents pathway names; the color represents the different *p* values. (**B**) Bubble plots of the enrichment analysis. The X-axis represents −log10 (*p* value), and the Y-axis represents pathway names; the size and color of the dots represent different enrichment ratios and *p* values, respectively.

To understand the biological significance and relevant metabolic pathways of the 48 identified candidate metabolites, we conducted metabolic pathway enrichment analysis. We observed that these 48 candidate metabolites were enriched in 10 different metabolic pathways (Supplementary Table S4). As shown in Figure 7, the metabolic pathways including fatty acid biosynthesis, cardiolipin biosynthesis, phosphatidylethanolamine biosynthesis, etc. Based on the *p* values, the 48 metabolites were significantly enriched in the fatty acid biosynthesis pathway. These results suggest that alterations in these metabolic pathways might account for the efficacy of inulin in preventing NAFLD.

## 3. Discussion

NAFLD is the most common chronic liver disease worldwide and has become a serious public health concern globally; however, its pathogenesis remains unclear, and there is yet no efficient clinical treatment. Hence, it is critical to explore efficient therapies for NAFLD. In the present study, we found that inulin could reduce body weight, improve serum biochemical indices, and restore histopathological changes caused by NAFLD in mouse livers. To determine the underlying mechanisms, we conducted a serum metabolomics study and obtained 48 candidate metabolites enriched in different metabolic pathways, a result that might account for the efficacy of inulin in preventing NAFLD.

Inulin is a flexible oligosaccharide and a versatile substance with several applications in food and pharmaceutical fields, and it has been implicated in the treatment of NAFLD. However, the mechanisms through which inulin prevents NAFLD remain unknown. It is known that the composition of serum metabolites may be changed in a state of disease [17]. Metabolic disturbance is a primary driver of the onset and progression of NAFLD. Thus far, several targeted and untargeted metabolomic studies have been conducted to identify metabolic biomarkers associated with the pathophysiology of NAFLD [18]. For instance, several serum metabolomics studies have shown that the plasma levels of glycocholate, taurocholate, and glycochenodeoxycholate are significantly elevated in NAFLD patients, showing that these molecules might be biomarkers of NAFLD [19]. Pharmaceutical drugs can also alleviate NAFLD/NASH by modulating serum metabolites and related metabolic pathways. Based on a serum metabolomics analysis, a previous study reported that vitamin E could alleviate high-fat-diet-induced NAFLD, and the underlying mechanism might be associated with the biosynthesis of cofactors, mainly pyridoxine and betaine [20]. Another study found that the effect of *delphinium brunonianum* extract on NASH might be correlated with the modulation of 22 serum metabolites, which are involved in several metabolic pathways (such as unsaturated fatty acid biosynthesis and the arachidonic acid metabolism pathway) [21]. In the present study, we report that inulin could augment serum biochemical indices and restore histopathological changes in mice with high-fat-diet-induced NAFLD. We also identified a batch of serum candidate metabolites (such as PS, DGLA, L-carnitine, and 13-HODE) enriched in different metabolic pathways, which might account for the efficacy of inulin in preventing NAFLD.

PS is a negatively charged phospholipid with a highly uneven distribution in cellular membranes, and it is essential for various biological processes in health and diseases (such as diabetes and liver diseases) [22,23]. It has been reported that PS plays a role in controlling insulin secretion, regulating insulin signaling transduction, and mediating the progression of diabetic complications [24]. Several studies have also implicated PS in the progression of liver diseases; however, little is known regarding the changes in serum PS abundance following the onset of NAFLD or NASH. According to a previous study, hepatic Mfn2 deficiency reduced PS transfer and phospholipid synthesis, leading to endoplasmic reticulum stress and the development of a NASH-like phenotype and liver cancer; this finding indicated that hepatic PS might be a protective factor against the development of advanced liver diseases [25]. Another study revealed that docosahexaenoic-acid-enriched phosphatidylserine (DHA-PS) increased the diversity and richness of beneficial intestinal microorganisms, which suggested that DHA-PS could be used as a dietary supplement and functional food to combat high-fat-diet-induced NAFLD. However, some studies have reported contrasting findings, with hepatic PS being thought to exacerbate the progression of hepatocellular carcinoma [26]. In the present study, we observed that the serum levels of several PSs, including PS (16:0/18:0) and PS (16:0/18:1(9Z)), were significantly higher in the high-fat-diet-induced NAFLD mice compared to those in the control group mice, and inulin treatment restored this change. Further studies are required to fully clarify the effect of different serum PS levels in NAFLD, constituting a finding that may enable the identification of potential therapies for NAFLD. We also observed a perturbance in the serum levels of DGLA in the NAFLD model group, and this change was restored by inulin treatment. 

DGLA is a 20-carbon omega-6 polyunsaturated fatty acid derived in vivo from the essential fatty acid linolenic acid, and it has recently emerged as a significant molecule for differentiating healthy from inflamed tissues [27,28]. Abundant evidence has revealed that altered serum DGLA levels are associated with many metabolic diseases, such as obesity, diabetes, and liver diseases. It has been reported that the serum levels of DGLA were significantly higher in overweight or obese individuals compared to those in healthy people [29]. A high serum DGLA level was associated with obesity, body fat accumulation, high ALT levels, and insulin resistance among patients with type 2 diabetes [30]. A cross-sectional study on liver diseases revealed that NAFLD subjects in a Japanese working population had higher serum levels of the phospholipid DGLA [31]. Matsuda et al. reported that a high serum DGLA level is significantly correlated with body mass index and homeostasis model assessment-insulin resistance (HOMA-IR) results, thus indicating that DGLA is a significant determinant and could be used as a predictive marker for hepatic steatosis [32]. In the present study, we observed that the serum DGLA level was higher in the model group than in the control group and lower in the inulin group than in the model group. These results are consistent with results from previous studies, which indicated that serum DGLA might play an important role in NAFLD progression and that inulin might prevent NAFLD partially by suppressing the serum DGLA level.

Apart from PS and DGLA, we also observed that the levels of some other serum metabolites (such as L-carnitine and 13-HODE) were affected in the model group and restored by inulin treatment. L-carnitine is a naturally occurring compound found in most mammalian tissues, and it plays a role in transporting activated long-chain fatty acids (long-chain fatty acyl-CoAs) into the mitochondria for degradation by β-oxidation [33]. Several studies have demonstrated that L-carnitine might play an important role in NAFLD progression. Reduced levels of L-carnitine might lower fatty acid oxidation and could function as a contributing factor in liver fat accumulation; moreover, supplementation with L-carnitine might reduce liver fat and liver ALT and AST levels in NAFLD patients [34]. A meta-analysis also revealed that L-carnitine supplementation decreased the levels of AST, ALT, TG, and HOMA-IR in individuals with NAFLD [35]. Our results are consistent with findings from previous studies to some extent. We observed that L-carnitine levels were decreased in NAFLD mice and increased after inulin treatment, which suggested that inulin might prevent NAFLD partially by increasing L-carnitine levels. A previous study reported that serum 13-HODE levels were significantly higher in NAFLD patients than in healthy individuals [36]. In another study, the levels of both serum and liver 13-HODE were increased in NAFLD rats. This finding suggests that 13-HODE could serve as a potential biomarker for NAFLD development [37]. In the present study, we found consistent results: serum 13-HODE levels were elevated in NAFLD mice and restored by inulin treatment. Overall, this finding indicates that the efficacy of inulin in preventing NAFLD might be associated with the modulation of serum 13-HODE levels.

Pathway enrichment analysis showed that the identified candidate metabolites were involved in 10 different metabolic pathways, and most metabolic pathways (such as fatty acid biosynthesis and cardiolipin biosynthesis) might be associated with NAFLD development. Reduced levels of β-oxidation, along with increased lipogenesis, result in lipid accumulation in hepatocytes; the subsequent production of reactive oxygen species and induction of a hepatocyte injury contribute to hepatic inflammation and fibrosis through the activation of Kupffer cells and hepatic stellate cells, leading to the development of NAFLD [38]. For instance, a previous study showed that the exposure of silica nanoparticles might enhance fatty acid synthesis and inhibit fatty acid β-oxidation and lipid efflux, accounting for the increased hepatic TC and TG levels observed in NAFLD progression [39]. Another study reported that cardiolipin abnormalities have been associated with mitochondrial dysfunction in several physiopathological conditions, including NAFLD [40]. In the present study, we observed that the target metabolites of inulin were enriched in many pathways; this finding indicates that inulin might prevent NAFLD partially through the regulation of related metabolic pathways such as fatty acid biosynthesis and cardiolipin synthesis.

Our present study has some limitations. First, the data were obtained via UPLC-MS metabolomics analysis, which identified many candidate metabolites and some related metabolic pathways. However, we did not perform additional analyses with functional experiments. Hence, we are unaware of how these metabolites precisely influence NAFLD progression. Further studies, including transcriptomics or proteomics analyses, are required to validate our findings, and additional investigations are needed to validate the function of the identified candidate metabolites. Second, our findings were obtained from limited animal models, and the performance of human-based studies is warranted to further verify the obtained data for subsequent clinical translation.

In summary, we developed animal models, observed the preventive effect of inulin on NAFLD, and performed a serum metabolomics analysis to reveal the underlying mechanisms regulating this preventive effect. The results suggest that inulin might prevent NAFLD by modulating the levels of serum candidate metabolites such as PS, DGLA, L-carnitine, and 13-HODE and their correlated metabolic pathways. Thus, our study may help us understand the efficacy of inulin in preventing NAFLD. Further investigations of the identified candidate metabolites may enable the development of novel therapies for NAFLD.

## 4. Materials and Methods

### 4.1. Experimental Animals

Thirty-six male C57BL/6J mice (6 weeks old, 12–14 g) were purchased from Shanghai SLAC Laboratory Animal Co. Ltd. (Shanghai, China), maintained in the Experimental Animal Centre of Shanghai University of Traditional Chinese Medicine, and kept under constant temperature and humidity conditions with a 12 h light/dark cycle. After one week of adaptive feeding, mice were randomly assigned to three groups. The control group was fed a normal chow diet (15.8% of kcal as fat, Research Diets, New Brunswick, NJ, USA), the NAFLD model group was fed a commercial D12492 high-fat diet (60% of kcal as fat; Research Diets, New Brunswick, NJ, USA), and the inulin group was fed a D12492 high-fat diet and 8% inulin (Fengning Ping’an Hi-Tech Industry Co., Ltd., Chengde, China). The mice had free access to sterile water during the experiment. After 8 weeks, mice in the three groups were fasted for 12 h, weighed, and injected with 10% chloral hydrate for anesthesia to collect samples. All animal experiment procedures were approved by the Animal Experiment Ethics Committee of Shanghai University of Traditional Chinese Medicine.

### 4.2. Biochemical Index Analysis

Blood samples were collected from the mice’s eyeballs into 1.5 mL centrifuge tubes and then placed in a sterile ice box for approximately 1 h. These samples were then centrifuged for 15 min at 3000 rpm and 4 °C using a mini centrifuge (Servicebio, Wuhan, China) to collect serum samples. Serum ALT, AST, TG, TC, HDL-C, and LDL-C levels were analyzed by using an automatic biochemical analyzer (Toshiba, Tokyo, Japan) in accordance with the manufacturer’s instructions.

### 4.3. Histopathological Analysis

The mice’s livers were removed and weighed, and the ratio of liver weight to body weight was calculated. Liver tissues were then cut into small pieces and repacked for further analysis. For H&E staining, liver tissues were fixed with 10% neutral formalin for one week, dehydrated, paraffin-embedded, sectioned into 4 μm thick slices, and stained with hematoxylin and eosin. The total NAFLD Activity Score (NAS score) was evaluated according to our previous study [41], which included three histological features, including steatosis (0–3), lobular inflammation (0–3), and hepatocellular ballooning (0–2). For ORO staining, liver tissues were frozen in an optimal-cutting-temperature compound (OCT, Sakura Tissue-Tek, Torrance, CA, USA); the tissues were then sectioned into 8 µm thick slices, stained with ORO, and counter-stained with hematoxylin. Finally, the slices of liver tissues were examined under a light microscope (Nikon, Tokyo, Japan) at ×200 magnification to observe the histopathological changes.

### 4.4. Serum Metabolomics Data Acquisition

Following serum collection, serum metabolomics analysis was performed at Shanghai WeiHuan Biotechnology Co., Ltd. (Shanghai, China), according to the manufacturer’s constructions. Briefly, 100 μL of each sample was removed, and 300 μL of methanol (containing 5 μg/mL of 2-chloro-L-phenylalanine as an internal standard) was added to it. The solutions were mixed using a vortex mixer for 1 min. The mixture was then centrifuged for 10 min at 13,000 rpm and 4 °C. The supernatant was transferred to sample vials for detection. In-house quality control samples were prepared by mixing an equal amount of each sample and passing it through a 0.45 μm filter membrane. LC-MS analysis was performed with an Agilent 1290 InfinityⅡUHPLC system coupled with an Agilent 6545 UHD and Accurate-Mass Q/TOF mass spectrometer. The following settings were used for the UPLC system: (1) chromatographic column—Waters X Select R HSS T3 (2.5 μm; 100 × 2.1 mm); (2) mobile phase—A: aqueous solution with 0.1% formic acid and B: acetonitrile solution with 0.1% formic acid; (3) flow rate—0.4 mL/min; (4) column temperature—40 °C; (5) injection volume—4 μL; and (6) optimized gradient elution condition—0–3 min, 20% B; 3–9 min, 20–95% B; 9–13 min, 95% B; 13–13.1 min, 95–5% B; and 13.1–16 min, 5% B. MS was performed in both positive and negative ion modes. The parameters were optimized as follows: (1) capillary voltage—4.5 kV in the positive mode and 3.5 kV in the negative mode; (2) drying gas flow—8 L/min in the positive mode and 10 L/min in the negative mode; (3) gas temperature—325 °C; (4) nebulizer pressure—20 PSIG; (5) fragmentor voltage—120 V; (6) skimmer voltage—45 V; and (7) mass range—*m*/*z* 50–1500.

Raw data were converted into the common (mzData) format using Agilent Masshunter Qualitative Analysis software version B.08.00 (Agilent Technologies, Santa Clara, CA, USA). In the R software (version 4.3.0) platform, the XCMS program was used for peak identification, retention time correction, and automatic integration pretreatment. The data were subjected to internal standard normalization (involving the division of the original peak area by the corresponding internal standard peak area). Visualization matrices containing sample name, *m*/*z*-RT pair, and peak area were obtained.

### 4.5. Reagents and Solvents

The main reagents and solvents used in the present study are listed as follows. Methanol and acetonitrile (HPLC grade) were purchased from Merck (Dannstadt, Germany). Formic acid (LC-MS grade) and 2-Chloro-L-phenylalanine (LC-MS grade) were purchased from Sigma-Aldrich (St. Louis, MO, USA). Distilled water was purchased from Watsons (Guangzhou, China), along with physiological saline (0.9% sodium chloride solution). Analytically pure isopropanol, absolute ethanol, xylene, n-butanol, and neutral gum were purchased from Sinopharm Chemical Reagent Co. (Shanghai, China). Dewaxing solution, 4% paraformaldehyde, hematoxylin–eosin (HE) staining kit, Oil Red O staining solution, hematoxylin staining solution, differentiation solution, Dako Bluing Buffer, and Glycerol Jelly Mounting Medium were purchased from Servicebio (Wuhan, China).

### 4.6. Metabolomics Data Analysis

To screen candidate metabolites, the metabolomics data were subjected to multivariate and univariate statistical analyses. For the multivariate statistical analysis, PCA and OPLS-DA models were constructed using SIMCA 14.1 software, and VIP values of OPLS-DA were obtained. Meanwhile, cross-validation analysis of variance (CV-ANOVA) was conducted to calculate *p* values for OPLS-DA models, and a permutation test conducted 200 times for OPLS-DA models was applied to obtain *R*^2^ and Q^2^ values to examine the validity and potential overfit. For univariate statistical analysis, an independent samples *t*-test was used, and *p* values between the groups were obtained. Fold changes of metabolites were calculated between the groups based on the ratio of the average normalized peak intensity. Differential metabolites between the groups were screened out based on the threshold values of VIP > 1, *p* < 0.05, and a number of fold changes >1.2 or <0.83. A Venn diagram (R package, version 1.6.2) was used to obtain the intersection of differential metabolites between the pairwise groups. Hierarchical clustering was performed to reveal the expression patterns of differential metabolites among the groups and visualize metabolites whose changes were reversed by inulin treatment. Pathway enrichment analysis was performed to identify biologically relevant patterns by using the online tool MetaboAnalyst 6.0 “https://www.metaboanalyst.ca/ (accessed on 17 June 2024)”.

### 4.7. Statistical Analysis

The measurement data were expressed as means ± standard deviation. By using SPSS 20.0, all data were statistically analyzed through one-way analysis of variance (ANOVA), followed by an independent samples *t*-test. A *p*-value of less than 0.05 was considered statistically significant.

## Figures and Tables

**Table 1:** List of 48 differential metabolites whose changes were reversed by inulin treatment.

Metabolites	Model vs. Control Fold Change	Model vs. Control *p* Value	Inulin vs. Model Fold Change	Inulin vs. Model *p* Value
(3b,16a,20R)-25-Acetoxy-3,16,20,22-tetrahydroxy-5-cucurbiten-11-one 3-glucoside	1.6	0.01	0.65	0.01
13-HODE	1.27	0.01	0.71	0
1-Stearoylglycerophosphocholine	1.25	0	0.68	0.02
2-Methylbutyrylglycine	0.74	0.01	1.49	0
3a,7a,12a-Trihydroxy-5b-cholestan-26-al	8.42	0	0.75	0.02
3alpha-Acetomethoxy-11alpha-oxo-12-ursen-24-oic acid	1.28	0	0.64	0
3-Hydroxycapric acid	2.07	0	0.74	0.01
3-Hydroxydodecanoic acid	2.19	0	0.74	0
3-Methylcytidine	1.35	0	0.72	0.01
3-Oxododecanoic acid	1.65	0.03	0.56	0
3-Oxohexadecanoic acid	1.68	0	0.79	0.01
3-Oxotetradecanoic acid	2.31	0	0.64	0
6-[(2-carboxyacetyl)oxy]-3,4,5-trihydroxyoxane-2-carboxylic acid	0.82	0	1.22	0.04
7-Hydroxyoctanoic acid	1.83	0	0.74	0
Cyclolinopeptide A	1.26	0	0.69	0.01
Cytidine triphosphate	0.8	0.01	1.25	0.01
DG(O-18:0/2:0/0:0)	16.85	0	0.67	0
Diethylcarbamazine N-oxide	1.76	0.01	0.56	0
Dihomo-gamma-linolenic acid	1.42	0	0.8	0.01
Diisobutyl adipate	4.37	0	0.81	0
Famotidine	1.39	0	0.69	0
Ganoderiol I	1.31	0	0.67	0.01
Guavin A	1.3	0	0.71	0.04
Hexadecanedioic acid	2.56	0	0.71	0.01
Humulinic acid A	6.49	0	0.8	0.01
Iohexol	0.66	0	1.57	0.02
Kudzusaponin SA4	1.4	0.02	0.73	0.02
L-Menthyl acetoacetate	1.95	0	0.58	0
LysoPC(18:0/0:0)	1.34	0	0.68	0.01
LysoPE(0:0/18:1(11Z))	1.83	0	0.81	0.02
Notoginsenoside I	0.48	0	1.79	0.02
Phenol sulphate	0.15	0	8.81	0
PS(16:0/18:0)	3.4	0	0.75	0.01
PS(16:0/18:1(9Z))	5.99	0	0.42	0.01
Rollinecin A	0.56	0	1.27	0
TG(14:0/20:1(11Z)/O-18:0)	0.63	0	1.56	0.02
TG(15:0/20:0/20:3(5Z,8Z,11Z))	0.74	0.01	1.51	0.02
TG(15:0/20:1(11Z)/O-18:0)	0.66	0	1.53	0.03
TG(15:0/20:2n6/O-18:0)	0.65	0	1.47	0.03
TG(18:0/18:0/18:0)	0.64	0	1.6	0.02
TG(20:0/24:1(15Z)/20:0)	2.58	0	0.63	0
TG(20:1(11Z)/24:0/20:1(11Z))	1.83	0	0.61	0
TG(22:0/20:3(5Z,8Z,11Z)/22:0)	1.76	0	0.64	0
TG(22:0/22:5(4Z,7Z,10Z,13Z,16Z)/22:0)	1.28	0	0.68	0
TG(22:0/22:6(4Z,7Z,10Z,13Z,16Z,19Z)/22:0)	1.4	0	0.63	0
TG(22:5(4Z,7Z,10Z,13Z,16Z)/22:6(4Z,7Z,10Z,13Z,16Z,19Z)/22:5(4Z,7Z,10Z,13Z,16Z))	2.01	0	0.53	0
TG(22:6(4Z,7Z,10Z,13Z,16Z,19Z)/22:5(4Z,7Z,10Z,13Z,16Z)/22:6(4Z,7Z,10Z,13Z,16Z,19Z))	1.93	0	0.55	0
YGM 5A	1.41	0	0.71	0.04

## Data Availability

Data is contained within the article or Supplementary Materials.

## Supplementary Materials

The following supporting information can be downloaded at https://www.mdpi.com/article/10.3390/ph17070895/s1. Table S1: List of 419 differential metabolites between the model and control groups; Table S2: List of 207 differential metabolites between the inulin and model groups; Table S3: List of 115 overlapped differential metabolites; Table S4: List of enriched metabolic pathways.
